# A new tool for assessing the cultural adaptation of cognitive tests: demonstrating the utility of the Manchester Translation Evaluation Checklist (MTEC) through the Mini-Mental State Examination Urdu

**DOI:** 10.1192/bjo.2022.620

**Published:** 2022-12-19

**Authors:** Nadine Mirza, Muhammed Wali Waheed, Waquas Waheed

**Affiliations:** Centre for Primary Care and Health Services Research, University of Manchester, UK; Leicester Medical School, University of Leicester, UK

**Keywords:** Cross cultural, diagnosis, psychometrics, scale development, transcultural

## Abstract

**Background:**

Low- and middle-income countries contribute to the majority of dementia and mild cognitive impairment cases worldwide, yet cognitive tests for diagnosis are designed for Western cultures. Language and cultural discrepancies mean that translated tests are not always reliable or valid. We propose a model for culturally adapting cognitive tests, one step of which is to assess the quality of any translation and cultural adaptation undertaken. We developed the Manchester Translation Evaluation Checklist (MTEC) to act as a tool for quality assessment and demonstrated its use by assessing a popular cognitive test that had been adapted.

****Aim**s:**

Assess quality of the translation and cultural adaptation of the Urdu Mini-Mental State Examination developed for a Pakistani population.

**Method:**

Two raters completed the MTEC for the Mini-Mental State Examination (MMSE) Urdu and compared feedback. All authors were fluent in English and Urdu and familiar with Pakistani culture.

**Results:**

Raters had 78.5% agreement across the MTEC. The MMSE Urdu was appropriately translated and retained grammar and verb tense, but three questions had spelling errors. Across 20 MMSE questions, 5 required further cultural adaptation because the questions were not understandable in daily use, comfortable to answer, relevant to the language and culture, and relevant to original concepts.

**Conclusions:**

The MTEC highlighted errors in the MMSE Urdu and demonstrated how this tool can be used to improve it. Future studies could employ the MTEC to improve existing translated measures of health assessment, particularly cognitive tests, and act as a quality check when developing new adaptations of tests and before psychometric validation.

There is an increased international demand for quick and accurate cognitive tests to facilitate the neuropsychiatric assessment process as instances of cognitive decline grow.^1^ Mild cognitive impairment (MCI) is present in 42% of the global population over 60 years of age^2^ and there are approximately 55 million people living with dementia globally, expected to rise to 139 million by 2050.^3^

The majority of cases are seen in low- and middle-income countries (LMICs), with dementia in these regions accounting for two-thirds of total global cases.^3,4^ These high rates of dementia and MCI found primarily in LMICs may be attributed to growing numbers of elderly people in these countries.^5^ Although the number of older people is estimated to increase over the next 15 years, by 71% in Latin America and the Caribbean, 66% in Asia and 64% in Africa, there is only a 41% expected increase in North America and 23% in Europe.^5^

Furthermore, LMICs such as India, China and countries in Latin America show higher rates of risk factors for dementia and MCI.^4^ These include a higher prevalence of depression, diabetes, hypertension and obesity,^4,6–8^ as well as living circumstances such as reduced education in early life, mid-life hearing loss, physical inactivity, smoking and social isolation.^4^ Research in high-income countries has determined that risk factors account for 35% of dementia cases but it is predicted that this number is actually higher when accounting for LMICs.^5^

Many countries in North America and Europe have also seen both increased economic and forced migration after the 1950s and 1970s, allowing them to form significant ethnic minority populations from LMIC backgrounds.^9,10^ Whereas in the past ethnic minorities were more likely to return to their country of origin as they grew older, owing to the growing disparity in human development between LMICs and high-income countries, many now choose to permanently settle in their old age in the country they have migrated to.^11^ Therefore, we now also see increasingly growing ageing ethnic minority populations in Western countries.^12^

These older people from ethnic minorities remain at risk for developing dementia and MCI owing to their retained susceptibility for risk factors found in LMICs that they share a heritage with.^12,13^ Furthermore, ethnic minorities in Western countries are more likely to come from a low socioeconomic background, which has been associated with developing dementia and MCI.^14,15^

## Language and cultural shortcomings of current cognitive tests

Cognitive tests, which are incorporated in both the screening and diagnosis of dementia and MCI, allow for early detection and accuracy in neuropsychiatric assessment.^16^ However, the majority of cognitive tests available at present are designed for English speakers in the context of Western cultures such as those in Europe and North America.^1,17,18^ They were standardised on male, White out-patients, not accounting for the populations of non-Western countries, including LMICs.^17,18^

Owing to the content of cognitive tests, both language and culture affects an individual's perception of test questions and responses to them. When these English-language cognitive tests are administered to non-English speakers and to those from Western cultural backgrounds, such as Europeans and North Americans, we see reduced sensitivity and specificity and higher rates of false-positive and false-negative scores for the non-native English speakers.^19–22^ Thus, there is a gap in the tests available to assess the cognitive health of culturally diverse populations, as well as populations in countries in the global East.

One proposed solution is to develop and pilot new cognitive tests but this is a time-consuming endeavour and not always feasible.^23^ An alternative is to adjust cut-off scores of existing cognitive tests for different language-speaking groups and cultural populations but this is criticised for reducing sensitivity, specificity and likelihood ratios.^24,25^

A third solution in practice has been to translate existing cognitive tests into target languages, with the use of the back-translation method.^26^ This involves translating a test directly into a target language, translating it back into the original language and comparing the old and new versions to see if there are any discrepancies.^26^ However, despite the rate at which back-translation is used, it still results in cognitive tests that produce false-positive and false-negative scores.^27^ This is because the process of changing a cognitive test from one language to another and then back-translating can result in words being mistranslated, questions developing different meanings and loss of the concepts being assessed.^26–28^

Furthermore, translation does not account for cross-cultural influence on the ability to answer questions beyond fluency in a target language.^1,27,28^ This is commonly seen in cognitive tests as they utilise questions requiring familiarity with the Western alphabet, names, idioms or history to assess cognitive domains such as language and memory.^21,27,28^ This cultural bias in test questions compromises the reliability of these cognitive tests:^29,30^ the same person may produce different scores owing to misunderstandings propagated by mismatched language and culture.^27^ The validity of these cognitive tests is also questionable: we are uncertain whether the results are due to the individual's state of health or due to errors in translation or differences in culture.^27^ Therefore, existing cognitive tests require not just translation but adaptation for different cultures.^29–31^

## A model for the translation and cultural adaptation of cognitive tests

Flaherty et al^28^ proposed that the first step in translating and culturally adapting any health measure is the selection process: literature is searched to see which tests may be suitable for a target population.^28,32^ A list of potential tests is drawn up and one test is hand-selected based on whether it has been used in cultural groups similar to the target population, whether it has been translated for several languages before and whether it has been psychometrically validated for diverse groups.^28,32^ There are also considerations beyond language and culture, such as the frequency of a test's use across published literature, the format it is delivered in and the cost of use due to copyright. Once a test has been selected according to this guidance it is determined whether it is available for the target population's language and cultural context. Owing to the need for cross-cultural health measures, if the selected test is not available in both the target language and cultural context then it requires translation and cultural adaptation. There may be instances where an adapted version of a test matches the target language but not the cultural context, or vice versa. In that instance further translating or culturally adapting that version of the test is appropriate as an attempt at adaptation has already been started.

As regards cognitive testing, our team demonstrated that, for every cognitive test, incorporation of literature on that test and feedback from adaptors of that test allows the development of a set of guidelines on culturally adapting each of that test's questions.^33^ These methods were illustrated through forming cultural adaptation guidelines for the Addenbrooke's Cognitive Examination Version III^33^ and we later replicated this for the Montreal Cognitive Assessment.^34^

Following this methodology, a cognitive test can be translated and culturally adapted for any language and cultural context using its guidelines.^33,34^ Such newly adapted tests can then be culturally validated, a process in which they are administered to healthy controls within the target population and a cognitive interviewing approach is undertaken.^35^ This determines whether the questions in the newly adapted version are both understandable and acceptable in those without cognitive impairment and ascertains that there is no bias in the language and cultural context that may cause poor performance.^35^ Finally, the new version of the cognitive test can undergo a psychometric validation in people with dementia or MCI within the target population.

**Fig. 1:**
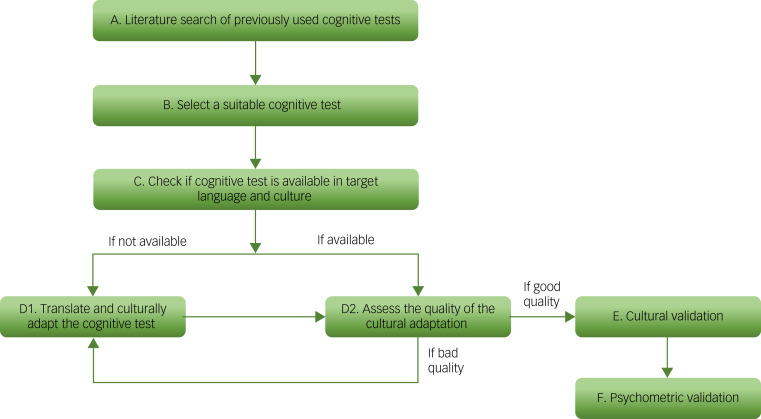
Proposed model for the translation and cultural adaptation of health measures in the context of cognitive testing.

We propose a translation and cultural adaptation model consisting of the above steps, which also incorporates not just selecting and culturally adapting an appropriate test, such as a cognitive test, but also assessing the quality of the adaptation (Fig. 1 outlines the model in the context of cognitive testing).

When selecting a cognitive test for a target population, finding one that has made accommodations for cultural context alone is not enough; quality assurance should be undertaken to determine whether the translation and cultural adaptation is of good quality. Similarly, if a suitable cognitive test is not available and one has to be translated and culturally adapted, a final quality assurance check can identify whether the translation or cultural adaptation needs to be changed or improved further. We therefore developed the Manchester Translation Evaluation Checklist (MTEC),^32^ a new tool for assessing the quality of the translation and cultural adaptation of questions in a health measure, to address this.

## Aims

In this paper we formally introduce the MTEC and demonstrate its utility as a quality assurance tool for the translation and cultural adaptation of health measures, particularly cognitive tests, by assessing an existing translation of a widely implemented cognitive test, the Mini-Mental State Examination (MMSE). The MMSE is a cognitive test that was developed to screen for both dementia and MCI.^36^ It consists of 13 overall questions, some with further subquestions, giving a total of 19 questions. It primarily assesses the cognitive domain of language, but also assesses orientation, registration, attention, memory and visuospatial abilities.^37^

The MMSE is one of the most commonly used cognitive tests globally. However, when translated and psychometrically validated in several non-English speaking countries, it demonstrated varied sensitivity and specificity owing to cultural biases^38–40^ and differing education levels.^41^ This is attributed to the heavy reliance on language-based tasks, which often require cultural adaptation beyond literal translation.^33^ Based on our proposed model, use of a quality assurance tool prior to psychometric validation may have highlighted where further translation and cultural adaptation was needed.

As it has universal application in the screening of dementia and MCI and many existing translations,^42^ the MMSE was an appropriate cognitive test for this demonstration. Given our own language and cultural background (we are all fluent in English and Urdu and are familiar with Pakistani culture), we utilised the MTEC to assess the quality of the translation and cultural adaptation of an Urdu version of the MMSE that had been translated for a Pakistani population.^42^

## Method

### Setting and ethics approval

This research took place at the Centre for Primary Care and Health Services Research at the University of Manchester, UK. This research did not require review or ethics approval.

### Materials

#### Manchester Translation Evaluation Checklist

The MTEC was developed by one of the authors (W.W.) at the University of Manchester to assess the quality of the translation and cultural adaptation of diagnostic health measures, particularly cognitive tests.^32^ Within the context of the MTEC, translation refers to whether the questions of a test were changed in terms of language, taking into account spelling, grammar, verb tense and retention of the original meaning of the question. Cultural adaptation refers to modifying or changing the question in terms of understanding, appropriateness and relevance to the cultural setting of the person being assessed, without changing the underlying concepts the question is assessing.

The MTEC was designed to account for Flaherty et al's five dimensions of cross-cultural equivalence, which reduce cultural bias in research instruments.^28^ These dimensions are technical, semantic, content, conceptual, and criterion equivalence, and should be retained by instruments, including cognitive tests, when they are translated and culturally adapted for different language and cultural backgrounds.^28^ When referring to cognitive tests, the dimensions would be defined as follows.^34^

- Technical equivalence: the test is administered in a manner that is culturally appropriate for the person being assessed.
- Semantic equivalence: after translation, the individual test questions retain the same meaning as their original versions.
- Content equivalence: after accounting for culture, the individual test questions are relevant to the culture of the person being assessed.
- Conceptual equivalence: after accounting for culture, the individual test questions still assess the same cognitive domains they were meant to at the original level of difficulty.
- Criterion equivalence: after accounting for culture, the overall test is still assessing for dementia or MCI.

The MTEC assesses whether a test, including cognitive tests, meets these equivalences, through a seven-query checklist (the MTEC queries are listed in the Appendix below and the full MTEC questionnaire with guidance on administration is given in Supplementary Material 1, available at https://doi.org/10.1192/bjo.2022.620). First, an original test question is input, translated into the target language and then back-translated. Following this, the seven queries are addressed to gather feedback on the quality of the test question's translation and cultural adaptation. Queries 1, 2 and 3, which are answered ‘yes’ or ‘no’, relate to the translation and address technical and semantic equivalence. Queries 4, 5, 6 and 7 are answered on a three-point Likert scale, with possible answers being ‘not acceptable’, ‘just acceptable’ and ‘acceptable’. They relate to the cultural adaptation and address content, criterion and conceptual equivalence. The amalgamation of this feedback shows any errors in translation or cultural adaptation that exist in the test.

#### MMSE Urdu

The MMSE Urdu had been translated using the back-translation method by researchers fluent in both English and Urdu for a Pakistani population.^42^ It was administered to 400 participants with varied literacy: 100 with dementia and 300 healthy controls.^42^ That study, which was the first validation study of the MMSE Urdu in Pakistan, found the MMSE Urdu to be a reliable and valid cognitive test in the screening of dementia in a Pakistani Urdu-speaking population. We contacted the corresponding author of the MMSE Urdu to acquire their version of the test, the original English version they translated and culturally adapted, and the publication on their MMSE Urdu's psychometric validation.^42^ Table 1 gives a comparison between the back-translated questions in the MMSE Urdu and the original English versions they based them on (see Supplementary Material 2 for the MMSE Urdu).

**Table 1:** The Mini-Mental State Examination (MMSE) and back-translated MMSE Urdu questions^42^

Cognitive domain	MMSE English questions	Back-translated MMSE Urdu questions
Orientation	Q1 What is the year?	Q1 What year is this?
Orientation	Q2 What is the season?	Q2 What season/weather is this?
Orientation	Q3 What is the date?	Q3 What date is this?
Orientation	Q4 What is the day?	Q4 What day is this?
Orientation	Q5 What is the month?	Q5 What month is this?
Orientation	Q6 Where are we? (state)	Q6 Which province are we in?
Orientation	Q7 Where are we? (country)	Q7 Which country are we in?
Orientation	Q8 Where are we? (hospital)	Q8 Which hospital are we in?
Orientation	Q9 Where are we? (floor)	Q9 Which floor/ward are we in?
Orientation		Q10 Which city are we in?
Registration	Q10 Name 3 objects.	Q11 Take the name of 3 objects.
Registration	Q11 Repeat the names of the 3 objects from Question 10.	Q12 Repeat the names of the 3 objects from Question 11.
Attention	Q12 Serial 7s. Alternatively, spell ‘WORLD’ backwards.	Q13 Serial 7s. Alternatively, spell ‘WORLD’ backwards or state the days of the week backwards.
Memory	Q13 Repeat the names of the 3 objects from Question 10.	Q14 Repeat the names of the 3 objects from Question 10.
Language	Q14 Indicate a pencil and watch.	Q15 Gesture towards a pencil and a watch.
Language	Q15 Repeat ‘No ifs, ands or buts’	Q16 Repeat ‘If and or but no’ or any other phrase.
Language	Q16 Follow a 3-stage command: ‘Take a paper in your hand, fold it in half and put it on the floor’	Q17 Follow a 3-stage command: ‘Take one paper in your right hand, fold it in half and put it on the floor’
Language	Q17 Read and obey: CLOSE YOUR EYES	Q18 Read and obey: CLOSE YOUR EYES
Language	Q18 Write a sentence.	Q19 Write a phrase of your choice.
Visuospatial abilities	Q19 Copy the design shown: 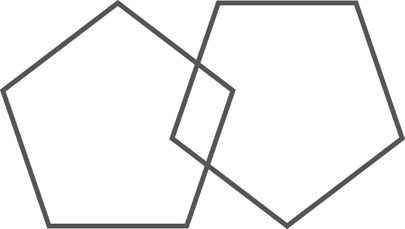	Q20 Copy this: 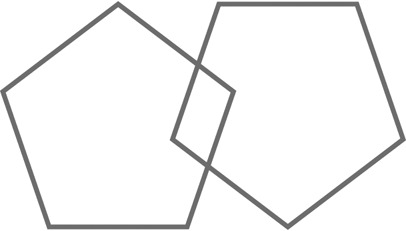

### Procedure

Two authors (N.M. and M.W.W.), a psychology postgraduate researcher and a medical student respectively, read through the MMSE Urdu. They then filled out an MTEC for each question, answering the queries using the guidance provided. They also qualitatively reflected on why they chose their answer for each query. The authors used a talk-aloud approach to compare this MTEC feedback and reasoning.^43^ When differences arose they were reviewed by a third author (W.W.), who is an experienced psychiatrist, until consensus was reached.

## Results

Initially, across the 20 MMSE Urdu questions with 7 MTEC queries each, N.M. and M.W.W. agreed on MTEC scoring 110 out of 140 times, resulting in a 78.5% agreement. After discussion with W.W. a consensus was achieved on all queries for all questions. The final MTEC results are shown in Table 2.

**Table 2:** The Manchester Translation Evaluation Checklist (MTEC) output for each question of the Mini-Mental State Examination (MMSE) Urdu

MMSE Urdu question	MTEC query						
	1	2	3	4	5	6	7
Q1	Yes	Yes	Yes	Acceptable	Acceptable	Acceptable	Acceptable
Q2	Yes	Yes	Yes	Acceptable	Acceptable	Acceptable	**Not acceptable**
Q3	Yes	Yes	**No**	Acceptable	Acceptable	Acceptable	Acceptable
Q4	Yes	Yes	**No**	Acceptable	Acceptable	Acceptable	Acceptable
Q5	Yes	Yes	Yes	Acceptable	Acceptable	Acceptable	Acceptable
Q6	Yes	Yes	**No**	Acceptable	Acceptable	Acceptable	Acceptable
Q7	Yes	Yes	Yes	Acceptable	Acceptable	Acceptable	Acceptable
Q8	Yes	Yes	Yes	Acceptable	Acceptable	Acceptable	Acceptable
Q9	Yes	Yes	Yes	Acceptable	Acceptable	Acceptable	Acceptable
Q10	Yes	Yes	Yes	Acceptable	Acceptable	Acceptable	**Not acceptable**
Q11	Yes	Yes	Yes	Acceptable	Acceptable	Acceptable	Acceptable
Q12	Yes	Yes	Yes	Acceptable	Acceptable	Acceptable	Acceptable
Q13	Yes	Yes	Yes	**Just acceptable**	**Just acceptable**	**Just acceptable**	**Not acceptable**
Q14	Yes	Yes	Yes	Acceptable	Acceptable	Acceptable	Acceptable
Q15	Yes	Yes	Yes	Acceptable	Acceptable	Acceptable	Acceptable
Q16	Yes	Yes	Yes	**Not acceptable**	**Just acceptable**	**Not acceptable**	**Not acceptable**
Q17	Yes	Yes	Yes	Acceptable	Acceptable	Acceptable	**Just acceptable**
Q18	Yes	Yes	Yes	Acceptable	Acceptable	Acceptable	Acceptable
Q19	Yes	Yes	Yes	Acceptable	Acceptable	Acceptable	Acceptable
Q20	Yes	Yes	Yes	Acceptable	Acceptable	Acceptable	Acceptable

### Quality of translation

All MMSE Urdu questions had been appropriately translated (Query 1) and their tense was retained (Query 2). MTEC Query 3 showed that in questions 3, 4 and 6, the Urdu word ‘*sahee*’, meaning ‘correct’, had been incorrectly spelled.

### Quality of cultural adaptation

Of the 20 MMSE Urdu questions, the MTEC found no issues in the cultural adaptation of 15 questions. Questions 1, 3–9, 11–12, 14–15 and 18–20 were found to be acceptable across all MTEC queries in the following ways:

- the wording of the questions was understandable and applicable for daily use in the Urdu-speaking Pakistani population (Query 4)
- Urdu-speaking Pakistanis being assessed would be comfortable responding to these questions (Query 5)
- the questions related to concepts relevant to the Urdu language and Pakistani culture (Query 6)
- the back-translation of the questions related to the same concepts as the original version of the questions (Query 7).

For the five MMSE Urdu questions that scored ‘just acceptable’ or ‘not acceptable’ on MTEC queries, the following reflective reasoning was demonstrated by the authors (N.M. and M.W.W.).

#### Question 2: What is the season?

This question received ‘not acceptable’ on Query 7 as the back-translation did not relate to the same concept as the original. The Urdu word for ‘season’ – ‘*mausum*’ – can also be directly translated as ‘weather’, depending on how the question is phrased. The translation in the MMSE Urdu, when back-translated, became ‘What weather is this?’. This Urdu translation would need rephrasing to ensure that the question conveys ‘season’, as opposed to ‘weather’.

#### Question 10: Where are we (city)?

This question received ‘not acceptable’ on Query 7 as it is not in the original version of the test, despite fulfilling other requirements of the MTEC.

#### Question 13: Spell ‘WORLD’ backwards (alternative to Serial 7s)

This question received ‘just acceptable’ on Queries 4, 5 and 6 as it asks for the English word ‘world’ to be spelled backwards. Since the majority of the Pakistani population are not fluent in English and there is a significantly low literacy rate, people may not find it easy to understand or comfortable to answer, and the concept does not relate to the Urdu language or Pakistani culture. Typically, this should result in a ‘not acceptable’ rating, however, in the MMSE Urdu another option is given if individuals are unable to spell ‘world’ backwards for reasons other than cognitive impairment. They could state the order of the days of the week in reverse and this addresses the issues mentioned above. Therefore, a combination of both these tasks results in a ‘just acceptable’ rating. The question received ‘not acceptable’ on Query 7 as the portion of it that asks to state the order of the days of the week in reverse is not in the original, despite fulfilling other requirements of the MTEC.

#### Question 16: Repeat the following – ‘no ifs, ands or buts’

This question received ‘not acceptable’ on Queries 4, 6 and 7 as the phrase ‘No ifs, ands or buts’ is not a saying in Urdu and it was presented as the literal translation of ‘ifs and or but no is’ in Urdu, which has no meaning. It would not be understandable and it is not relevant in Urdu or to Pakistani culture. As the purpose of this question is to see whether the individual will repeat a well-known phrase, the back-translation of this question shows that this concept is not retained. Therefore, ratings of ‘not acceptable’ were given. However, to address these issues in the MMSE Urdu an alternative was given that allows the administrator to state any phrase which could then be repeated by the participant. This received a rating of ‘just acceptable’ for Query 5, as repeating any phrase could be comfortable for an individual as long as it was in their target language. But as it is an addition made to the MMSE Urdu and not justified within the publication, it cannot be related to any original concept.

#### Question 17: Follow a three-stage command: ‘take a paper in your hand, fold it in half and put it on the floor’

This question received ‘just acceptable’ on Query 7 as the back-translation of the Urdu version asks the individual to use their right hand specifically, despite this not being in the original version of the test.

## Discussion

There is currently an international need for cognitive tests designed to assess for neuropsychiatric conditions such as dementia and MCI in individuals in LMICs or in ethnic minority populations in high-income countries.^17,18^ Such cognitive tests must account for the language, cultural background and specific understanding of individuals being assessed, to avoid bias in performance.^19–22^

We proposed a model for how existing cognitive tests could be translated and culturally adapted for a target population and a crucial step in that model is assessing the quality of the cultural adaptation being produced (Fig. 1). This quality assurance should act as a check to ensure that a test not only accounts for the language and culture of the person being assessed, but also maintains the integrity of the concepts being assessed. The MTEC does this by highlighting any errors in existing translations or cultural adaptations of a cognitive test, and allows for further improvements. We demonstrated this by using the MTEC to assess the quality of the translation and cultural adaptation of the MMSE Urdu.

As regards translation, the MMSE Urdu contained spelling errors but retained verb tense and had been appropriately translated overall. From the perspective of cultural adaptation, 5 out of 20 questions demonstrated several problems. These questions pertained to the cognitive domains of orientation and language, which are heavily reliant on cultural influence.^33,34^ As the MMSE Urdu was developed through a back-translation method^42^ the development process would not have accounted for cultural bias and performance beyond fluency in Urdu.^26–28^ This may explain why raters marked these questions as ‘just acceptable’ or ‘not acceptable’ on the MTEC.

Question 2, which assesses orientation, does not account for the word for ‘season’ also meaning ‘weather’ in Urdu. By avoiding a literal verbatim translation and engaging in nuanced cultural adaptation, rephrasing the question to ‘Which of the four seasons is it?’ would ensure that ‘season’ could not be confused for ‘weather’ in the Urdu question, while still retaining the original concept of the question.

Similarly, Question 13 for language (Question 12 in English), which asks Urdu speakers to spell the English word ‘WORLD’ backwards, demonstrates both a language and cultural mismatch, with Query 3 ‘just acceptable’ and Query 1 ‘not acceptable’. It attempts to mitigate this by offering a culturally appropriate alternative – stating the days of the week in reverse order. However, the task itself is already an alternative for the Serial 7s task, which individuals being assessed may not have the literacy requirement to complete.^42^ Instead of offering two alternatives to the Serial 7s task, a culturally appropriate MMSE Urdu might remove the ‘WORLD’ task and retain stating the days of week backwards as the only alternative.

Question 16 for language (Question 15 in English) had the most problems in terms of cultural adaptation, with Query 3 ‘not acceptable’ and Query 1 ‘just acceptable’. It is another example of a literal verbatim translation, where back-translation from one language to another has removed meaning from the phrase completely. As idioms and phrases are not necessarily universal it would have been more appropriate to have selected one within the cultural context of Pakistan, as opposed to random selection. To rectify this there is an option allowing the administrator to choose one themselves. However, the length of the phrase when spoken, the frequency of its use in the cultural setting, and the number of words and syllables within it all have psychometric significance in the validity of cognitive tests when assessing language ability. Therefore, idioms and phrases should not be selected at random by each assessor.

In Question 17 for language (Question 16 in English) the individual is asked to take a paper in their right hand as opposed to any hand. This may be accounting for cultural context because in Pakistan there is a preference for using the right hand, which is often encouraged and associated with cleanliness and purity. But no justification for this change has been provided in the publication detailing the development of the MMSE Urdu. Additionally, this does not mitigate the issues that may occur for those who are left handed and may instinctively use their dominant hand. By prioritising the right hand, the question's conceptual equivalence may be compromised.

Overall, we found that despite being psychometrically validated and currently in use, the MMSE Urdu still possesses errors in translation and potential for cultural bias for a wider Pakistani population. Therefore, this cognitive test would have benefited from a quality check with the MTEC beforehand to detect and amend these problems.

The MTEC allows for reflection on where a health measure, such as a cognitive test, currently has gaps in terms of its language and culture when it applies to a specific group, even if that test is currently in circulation. We have purposely ensured that the MTEC does not provide a quantitative score for judging whether the translation or cultural adaptation is deemed to be understandable and appropriate; there is no cut-off score at which a test is or is not suitable. This is because even a single error in translation or cultural adaptation can result in an overall compromised test. We can see from our demonstration with the MMSE Urdu that even one of the errors could potentially affect an individual's performance. Instead, the MTEC presents feedback and qualitative reflection that allow raters, whether they be reviewers such as in this instance or adaptors in the future, to consider their responses and utilise them to improve the quality. This ensures more standardisation across translated and culturally adapted versions of the same test.

### Limitations and future research

We acknowledge that raters must be familiar with the target language and culture of the desired population to be able to use the MTEC. However, this is likely to be expected of any research team when translating a test and, in fact, we would advocate this to ensure a more robust cultural adaptation. We have shown that the MTEC is suitable and easy to use for those who may not have clinical experience or extensive knowledge of psychometrics, and this expands its potential beyond research team members for use by non-experts in target populations. In studies where cultural adaptation of a test is undertaken as part of the methods, the MTEC could be administered by non-experts as a form of patient and public involvement, and in the role of co-producers.

We acknowledge that in this particular illustration we are limited by only having two raters provide feedback on the MMSE Urdu, despite high agreement. However, this still gave enough feedback to highlight key problems and allowed for a demonstration of the MTEC. In future we recommend a more diverse range of raters, which would result in more extensive and nuanced feedback.

Although future studies may wish to identify errors in existing cultural adaptations of cognitive tests as we have done, the MTEC can also be used by research teams in the development of new translations and cultural versions of tests. We propose repeatedly using the MTEC and making revisions in accordance with its feedback at various stages of scale development; this can be done by both researchers and co-producers. This would facilitate the making of tests for populations in LMICs and for ethnic minority groups in high-income countries.

As detailed in our model, the MTEC can also be used as a precursor to psychometric validation studies of cognitive tests in diverse populations to eliminate any remaining cultural bias. As the design of the MTEC has been theoretically influenced by Flaherty et al's principles of translating and culturally adapting health measures,^28^ future studies should also use the MTEC to assess the quality of other culturally adapted health measures beyond cognitive tests.

## Data Availability

The data that support the findings of this study are available from the corresponding author (N.M.) on reasonable request.

## Supplementary material

For supplementary material accompanying this paper visit http://doi.org/10.1192/bjo.2022.620. click here to view supplementary material

## Appendix

The Manchester Translation Evaluation Checklist (MTEC):^32^

- Query 1 Has the question been translated completely?
- Query 2 Has correct grammar and tense been used?
- Query 3 Have correct spellings been used?
- Query 4 Are the words used easily understandable and of daily use?
- Query 5 Would the responder be comfortable at answering the question?
- Query 6 Are the concepts used relevant to local language and population?
- Query 7 Does back-translation relate to same concepts as in original text?

## References

[1] Tanveer S, Croucher MJ, Porter R. Cultural modification of neuropsychiatric assessment: complexities to consider. BJPsych Open 2022; 8(2): e68. 35287781 10.1192/bjo.2022.33 PMC8935941

[2] Hu C, Yu D, Sun X, Zhang M, Wang L, Qin H. The prevalence and progression of mild cognitive impairment among clinic and community populations: a systematic review and meta-analysis. Int Psychogeriatr 2017; 29: 1595–608. 28884657 10.1017/S1041610217000473

[3] World Health Organization. Dementia. WHO, 2021 (https://www.who.int/en/news-room/fact-sheets/detail/dementia [accessed 8 Jan 2022]).

[4] Mukadam N, Sommerlad A, Huntley J, Livingston G. Population attributable fractions for risk factors for dementia in low-income and middle-income countries: an analysis using cross-sectional survey data. Lancet Glob Health 2019; 7: e596–603. 31000129 10.1016/S2214-109X(19)30074-9 PMC7617123

[5] Ferri CP, Jacob KS. Dementia in low-income and middle-income countries: different realities mandate tailored solutions. PloS Med 2017; 14(3): e1002271. 28350797 10.1371/journal.pmed.1002271 PMC5370095

[6] Adelman S, Blanchard M, Rait G, Leavey G, Livingston G. Prevalence of dementia in African–Caribbean compared with UK-born White older people: two-stage cross-sectional study. Br J Psychiatry 2011; 199: 119–25. 21653946 10.1192/bjp.bp.110.086405

[7] Gholap N, Davies M, Patel K, Sattar N, Khunti K. Type 2 diabetes and cardiovascular disease in South Asians. Prim Care Diabetes 2011; 5: 45–56. 20869934 10.1016/j.pcd.2010.08.002

[8] Seabrooke V, Milne A. Culture and Care in Dementia: A Study of the Asian Community in North West Kent. Alzheimer's and Dementia Support Services, 2004.

[9] Castelli F. Drivers of migration: why do people move? J Travel Med 2018; 25(1): tay040. 10.1093/jtm/tay040 30053084

[10] Van Mol C, Valk HD. Migration and immigrants in Europe: a historical and demographic perspective. In Integration Processes and Policies in Europe: Contexts, Levels and Actors (eds B Garcés-Mascareñas, R Penninx): 31–55. Springer, 2016.

[11] Mukadam N, Cooper C, Livingston G. A systematic review of ethnicity and pathways to care in dementia. Int J Geriatr Psychiatry 2011; 26: 12–20. 21157846 10.1002/gps.2484

[12] Richards M, Brayne C, Dening T, Abas M, Carter J, Price M, Cognitive function in UK community-dwelling African Caribbean and white elders: a pilot study. Int J Geriatr Psychiatry 2000; 15: 621–30. 10918343 10.1002/1099-1166(200007)15:7<621::aid-gps164>3.0.co;2-4

[13] Chui HC, Gatz M. Cultural diversity in Alzheimer disease: the interface between biology, belief, and behavior. Alzheimer Dis Assoc Disord 2005; 19: 250–5. 16327354 10.1097/01.wad.0000190802.03717.20

[14] Yaffe K, Falvey C, Harris TB, Newman A, Satterfield S, Koster A, Effect of socioeconomic disparities on incidence of dementia among biracial older adults: prospective study. BMJ 2013; 347: f7051. 10.1136/bmj.f7051 PMC3898154 24355614

[15] Fischer C, Yeung E, Hansen T, Gibbons S, Fornazzari L, Ringer L, Impact of socioeconomic status on the prevalence of dementia in an inner city memory disorders clinic. Int Psychogeriatr 2009; 21: 1096–104. 19712540 10.1017/S1041610209990846

[16] Panegyres PK, Berry R, Burchell J. Early dementia screening. Diagnostics 2016; 6(1): 6. 26838803 10.3390/diagnostics6010006 PMC4808821

[17] Tuerk R, Sauer J. Dementia in a Black and minority ethnic population: characteristics of presentation to an inner London memory service. BJPsych Bull 2015; 39: 162–6. 26755947 10.1192/pb.bp.114.047753 PMC4706140

[18] Fillenbaum GA, Heyman A, Williams K, Prosnitz B, Burchett B. Sensitivity and specificity of standardized screens of cognitive impairment and dementia among elderly black and white community residents. J Clin Epidemiol 1990; 43: 651–60. 2370572 10.1016/0895-4356(90)90035-n

[19] Regan JL. Ethnic minority, young onset, rare dementia type, depression: a case study of a Muslim male accessing UK dementia health and social care services. Dementia 2016; 15: 702–20. 24858552 10.1177/1471301214534423

[20] Weimer DL, Sager MA. Early identification and treatment of Alzheimer's disease: social and fiscal outcomes. Alzheimer Dement 2009; 5: 215–26. 10.1016/j.jalz.2009.01.028 PMC2785909 19362885

[21] Parker C, Philp I. Screening for cognitive impairment among older people in black and minority ethnic groups. Age Ageing 2004; 33: 447–52. 15217776 10.1093/ageing/afh135

[22] Khan F, Tadros G. Complexity in cognitive assessment of elderly British minority ethnic groups: cultural perspective. Dementia 2014; 13: 467–82. 24339067 10.1177/1471301213475539

[23] Beaton DE, Bombardier C, Guillemin F, Ferraz MB. Guidelines for the process of cross-cultural adaptation of self-report measures. Spine 2000; 25: 3186–91. 11124735 10.1097/00007632-200012150-00014

[24] Bohnstedt M, Fox PJ, Kohatsu ND. Correlates of mini-mental status examination scores among elderly demented patients: the influence of race-ethnicity. J Clin Epidemiol 1994; 47: 1381–7. 7730847 10.1016/0895-4356(94)90082-5

[25] Milani SA, Marsiske M, Cottler LB, Chen X, Striley CW. Optimal cutoffs for the Montreal Cognitive Assessment vary by race and ethnicity. Alzheimers Dement 2018; 10: 773–81. 10.1016/j.dadm.2018.09.003 PMC6247398 30505927

[26] Yu DS, Lee DT, Woo J. Issues and challenges of instrument translation. West J Nurs Res 2004; 26: 307–20. 15068554 10.1177/0193945903260554

[27] Wang WL, Lee HL, Fetzer SJ. Challenges and strategies of instrument translation. West J Nurs Res 2006; 28: 310–21. 16585807 10.1177/0193945905284712

[28] Flaherty JA, Gaviria FM, Pathak D, Mitchell T, Wintrob R, Richman JA, Developing instruments for cross-cultural psychiatric research. J Nerv Ment Dis 1988; 76: 257–63. 3367140

[29] Van de Vijver F, Tanzer NK. Bias and equivalence in cross-cultural assessment: an overview. Eur Rev Appl Psychol 2004; 54: 119–35.

[30] Vijver FV. Meta-analysis of cross-cultural comparisons of cognitive test performance. J Cross Cult Psychol 1997; 28: 678–709.

[31] Guillemin F, Bombardier C, Beaton D. Cross-cultural adaptation of health-related quality of life measures: literature review and proposed guidelines. J Clin Epidemiol 1993; 46: 1417–32. 8263569 10.1016/0895-4356(93)90142-n

[32] Waheed W. Prevalence and persistence of depression in Pakistani and white European in the United Kingdom [dissertation]. University of Manchester, 2010.

[33] Waheed W, Mirza N, Waheed MW, Malik A, Panagioti M. Developing and implementing guidelines on culturally adapting cognitive tests: a qualitative illustration with the Addenbrooke's Cognitive Examination Version III (ACE-III). BMC Psychiatry 2020; 20(1): 492. 10.1186/s12888-020-02893-6 PMC7539399 33023520

[34] Khan G, Mirza N, Waheed W. Developing guidelines for the translation and cultural adaptation of the Montreal Cognitive Assessment (MoCA): a scoping review and qualitative synthesis. BJPsych Open 2022; 8(1), e21. 10.1192/bjo.2021.1067 PMC8811786 34991771

[35] Mirza N, Panagioti M, Waheed W. Cultural validation of the Addenbrooke's Cognitive Examination Version III Urdu for the British Urdu-speaking population: a qualitative assessment using cognitive interviewing. BMJ Open 2018; 8(12): e021057. 10.1136/bmjopen-2017-021057 PMC6303692 30552243

[36] Folstein MF, Folstein SE, McHugh PR. “Mini-mental state”: a practical method for grading the cognitive state of patients for the clinician. J Psychiatr Res 1975; 12(3): 189–98. 1202204 10.1016/0022-3956(75)90026-6

[37] Larner AJ. Manual of Screeners for Dementia: Pragmatic Test Accuracy Studies. Springer Nature, 2020: 51–4.

[38] Vas CJ, Pinto C, Panikker D, Noronha S, Deshpande N, Kulkarni L, Prevalence of dementia in an urban Indian population. Int Psychogeriatr 2001; 13: 439–50. 12003250 10.1017/s1041610201007852

[39] Prince M, Acosta D, Chiu H, Scazufca M, Varghese M, 10/66 Dementia Research Group. Dementia diagnosis in developing countries: a cross-cultural validation study. Lancet 2003; 361: 909–17. 12648969 10.1016/S0140-6736(03)12772-9

[40] Espino DV, Lichtenstein MJ, Palmer RF, Hazuda HP. Ethnic differences in Mini-Mental State Examination (MMSE) scores: where you live makes a difference. J Am Geriatr Soc 2001; 49: 538–48. 11380745 10.1046/j.1532-5415.2001.49111.x

[41] De Yébenes MJ, Otero A, Zunzunegui MV, Rodríguez-Laso A, Sánchez-Sánchez F, Del Ser T. Validation of a short cognitive tool for the screening of dementia in elderly people with low educational level. Int J Geriatr Psychiatry 2003; 18: 925–36. 14533125 10.1002/gps.947

[42] Awan S, Shahbaz N, Akhtar SW, Ahmad A, Iqbal S, Ahmed S, Validation study of the Mini-Mental State Examination in Urdu language for Pakistani population. Open Neurol J 2015; 9: 53–8. 26191094 10.2174/1874205X01509010053 PMC4503826

[43] Davison GC, Vogel RS, Coffman SG. Think-aloud approaches to cognitive assessment and the articulated thoughts in simulated situations paradigm. J Consul Clin Psychol 1997; 65(6): 950–8. 10.1037//0022-006x.65.6.950 9420356

