# Supplementary material for: A new tool for assessing the cultural adaptation of cognitive tests: demonstrating the utility of the Manchester Translation Evaluation Checklist (MTEC) through the Mini-Mental State Examination Urdu
Source: BJPsych Open. 2022 Dec 19;9(1):e5. doi: 10.1192/bjo.2022.620 (PMC9798223; doi:10.1192/bjo.2022.620)
Supplement: Supplementary file 1 [file bjosup.zip › S2056472422006202sup001.docx]

**
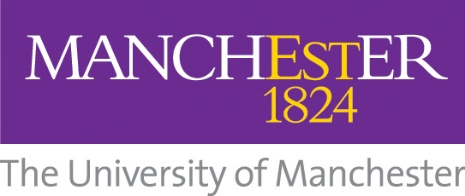
**

**MTEC Manchester Translation Evaluation Checklist**

Original question:

Translation:

Back translation:

| Q.1 Has the question been translated completely? | Yes | No |
| --- | --- | --- |
|  |  |  |
| Q.2 Has the correct grammar and tense been used? | Yes | No |
|  |  |  |
| Q.3 Have correct spellings been used? | Yes | No |
|  |  |  |

| Q.4 Are the words used easily understandable and of daily use? | Not Acceptable | Just Acceptable | Acceptable |
| --- | --- | --- | --- |
|  |  |  |  |
| Q.5 Would the responder be comfortable answering the question? | Not Acceptable | Just Acceptable | Acceptable |
|  |  |  |  |
| Q.6 Are the concepts used relevant to local language and population? | Not Acceptable | Just Acceptable | Acceptable |
|  |  |  |  |
| Q.7 Does back translation relate to the same concepts as the original text? | Not Acceptable | Just Acceptable | Acceptable |

**
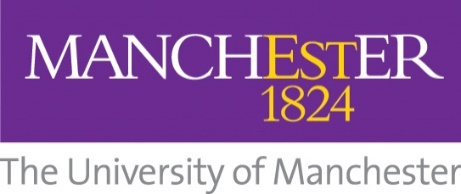
**

**Manchester Translation Evaluation Checklist Guidance**

1. ***Has the original English question been translated completely?***

The question must convey ALL the ideas and concepts present in the original version.

***2.* *Has the translation used the correct grammar and tense?***

Has the question been adjusted to account for grammar? Does the tense used in the translation still match that in the original exactly? eg. “Have you been feeling unwell?” is not the same as “Have you felt unwell?”.

1. ***Has the translation used the correct spelling?***
2. ***Would you be comfortable answering the original question?***

Is the question that is being asked culturally appropriate? In other words, is the question being asked acceptable to the target population’s cultural background? Would patients be willing to answer such a question?

1. ***How would you rate the translation’s choice of words? Are they easily understandable and of daily use?***

Are the individual words used in the translation accurate, and comprehensible to people of all social backgrounds and levels of education? Obscure and overly formal language should be avoided as far as possible.

1. ***In your opinion, are the concept(s) in the translation relevant to the local language and population?***

Are the concepts in the translation familiar to the culture? Are they easily understandable to the target population?

1. ***How well do the concept(s) in the translation relate to the concept(s) in the original English question?***

Does the translation “read” the same as the original? There should be no real difference in meaning between the two versions, and a person answering the questions in both languages should give the same response if the translation is accurate.
