# Supplementary material for: A new tool for assessing the cultural adaptation of cognitive tests: demonstrating the utility of the Manchester Translation Evaluation Checklist (MTEC) through the Mini-Mental State Examination Urdu
Source: BJPsych Open. 2022 Dec 19;9(1):e5. doi: 10.1192/bjo.2022.620 (PMC9798223; doi:10.1192/bjo.2022.620)

The Mini-Mental State Exam (MMSE)

Patient ______________________ Examiner ______________________ Date ___/___/_____

Orientation

	Score	Maximum
What is the (year) (season) (date) (day) (month)?	( )	5
Where are we (state) (country) (hospital) (floor)?	( )	5

Registration

	Score	Maximum
Name 3 objects : 1 second to say each. Then ask the patient all 3 after you have said them. Give one point for each correct answer. Then repeat them until he/she learns all 3. Count trials and record.	( )	3

Trials _______________

Attention and Calculation

	Score	Maximum
Serial 7's. 1 point for each correct answer. Stop after 5 answer. Althernatively spell "world" backward.	( )	5

Recall

	Score	Maximum
Ask for the 3 objects repeatd above. Give 1 point for each correct answer.	( )	3

Language

	Score	Maximum
Name a pencil and watch.	( )	2
Repeate the following "No ifs, ands, or buts"	( )	1
Follow a 3-stage command:	( )	3
"Take a paper in your hand, fold it in half, and put it on the floor."		
Read and obey the following: CLOSE YOUR EYES	( )	1
Write a sentence.	( )	1
Copy the design shown.	( )	1

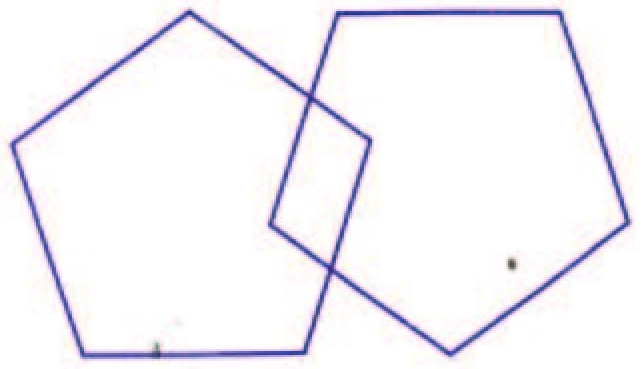

Total Score

ASSESS level of consciousness along a continum ______________ ______________

Alert Drowsy Stupor Coma

24-30: within normal limits: ≤ 23: cognitive impairment
(further formal testing recommended)

مینی مینٹل سٹیٹ ایگزامینیشن (MMSE)

مریض ____________ نگران ____________ تاریخ ______ \ \ ______

1۔ پہچان

یہ کونسا (سال ہے) (موسم ہے) (تاریخ ہے) (مہینہ ہے) (دن ہے) 5

کونسے (صوبے میں) (ملک میں) (قصبے/شہر میں) (ہسپتال میں) (کونسی منزل/وارڈ میں) 5

2۔ رجسٹریشن (ذہن نشینی)

تین چیزوں کے نام لیں (ہر ایک نام لینے کیلئے ایک سیکنڈ کا وقفہ لیں) پھر مریض سے کہیں کے یہ تینوں نام دہرائے۔

ہر صحیح جواب کا ایک نمبر دیں 3

جب تک مریض تینوں نام یاد نہ کرے جواب دہرائیں۔

3۔ توجہ اور حساب (جمع اور تفریق)

7 کی ترتیب، ہر صحیح جواب کا ایک نمبر لگائیں، پانچ جوابات کے بعد رک جائیں۔

متبادل ''World'' کے الٹے ہجے بتائیں؟ یا ہفتے کے دنوں کے نام الٹی ترتیب سے بتائیں؟

پانچ جوابات کے بعد رک جائیں 5

4۔ یاداشت

سوال نمبر 2 میں یاد کردہ تین اشیاء کے نام پوچھیں، ہر صحیح جواب کا ایک نمبر لگائیں 3

5۔ زبان

پنسل اور گھڑی کی طرف اشارہ کریں۔

آپ کے اشارے کرنے پر کیا مریض ان کے نام بتا سکتا ہے؟ 2

''اگر'' ''اور'' ''یا'' ''مگر نہیں'' ہے 1

(یا کوئی بھی فقرہ بول دیں)

6۔ کیا مریض ''تین مرحلوں'' کے حکم پر عمل کر سکتا ہے؟

اپنے دائیں ہاتھ میں ایک کاغذ پکڑیں۔ اسے دہرا تہہ کریں۔

کاغذ کو فرش پر رکھیں۔ 3

7۔ کیا مریض مندرجہ ذیل پڑھ کر عمل کر سکتا ہے؟ ''اپنی آنکھیں بند کر لیں''

(بڑے حروف میں لکھیں) 1

8۔ کیا مریض اپنی مرضی کا کوئی فقرہ لکھ سکتا ہے؟

فقرے میں فاعل اور مفعول کو ہونا ضروری ہے اور یہ بھی ضروری ہے کہ فقرہ معقول ہو (سمجھ میں آئے) 1

9۔ مندرجہ ذیل کو اس طرح بڑا کریں کہ اس کی ہر سائیڈ 1.5 انچ ہو

اور مریض سے کہیں کے اس کی نقل کریں 1

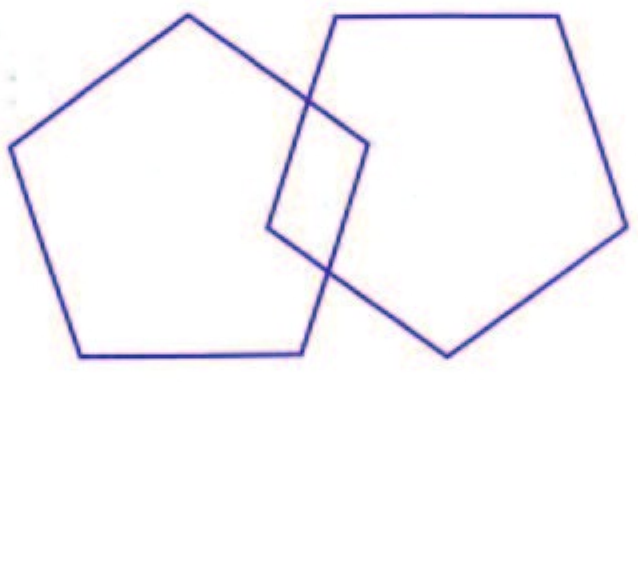

Supplement: Supplementary file 1 [file bjosup.zip › S2056472422006202sup002.pdf]
